# GM-CSF Monocyte-Derived Cells and Langerhans Cells As Part of the Dendritic Cell Family

**DOI:** 10.3389/fimmu.2017.01388

**Published:** 2017-10-23

**Authors:** Manfred B. Lutz, Herbert Strobl, Gerold Schuler, Nikolaus Romani

**Affiliations:** ^1^Institute for Virology and Immunobiology, University of Würzburg, Würzburg, Germany; ^2^Institute of Pathophysiology and Immunology, Medical University of Graz, Graz, Austria; ^3^Department of Dermatology, University Hospital Erlangen, Erlangen, Germany; ^4^Department of Dermatology, Venereology and Allergology, Medical University of Innsbruck, Innsbruck, Austria

**Keywords:** dendritic cells, GM-CSF, monocytes, Langerhans cells, macrophages

## Abstract

Dendritic cells (DCs) and macrophages (Mph) share many characteristics as components of the innate immune system. The criteria to classify the multitude of subsets within the mononuclear phagocyte system are currently phenotype, ontogeny, transcription patterns, epigenetic adaptations, and function. More recently, ontogenetic, transcriptional, and proteomic research approaches uncovered major developmental differences between Flt3L-dependent conventional DCs as compared with Mphs and monocyte-derived DCs (MoDCs), the latter mainly generated *in vitro* from murine bone marrow-derived DCs (BM-DCs) or human CD14^+^ peripheral blood monocytes. Conversely, *in vitro* GM-CSF-dependent monocyte-derived Mphs largely resemble MoDCs whereas tissue-resident Mphs show a common embryonic origin from yolk sac and fetal liver with Langerhans cells (LCs). The novel ontogenetic findings opened discussions on the terminology of DCs versus Mphs. Here, we bring forward arguments to facilitate definitions of BM-DCs, MoDCs, and LCs. We propose a group model of terminology for all DC subsets that attempts to encompass both ontogeny and function.

## Introduction

Dendritic cells (DCs) are major players to direct adaptive immunity or tolerance. More recently, the origins and possible subdivisions into DC subsets and the DC commonalities with macrophages (Mphs) have been discussed by numerous papers and reviews (1–7).

In their peripheral tissue-resident state, DCs act as immune sensors that recognize pathogens and then convert into a mature or activated state enabling their migration to the draining lymph node to stimulate T cell immunity (8). By contrast, during homeostasis lymphatic organ-resident DCs and steady-state migratory DCs contribute to immune tolerance (9).

This functional capacity of antigen transport from the periphery to the lymph nodes has been one of their major cellular characteristics distinguishing them from Mphs. Marked differences between DCs and Mphs have recently also been observed by quantitative proteomic analyses that point out differential and specific epigenetic programming of each cell type (10) or, at a more functional level, by dissecting and defining differential cellular mechanisms in endocytic recycling pathways of MHC I molecules for cross-presentation (11).

Several data indicate that murine bone marrow-derived DCs (BM-DCs), human monocyte-derived DCs (MoDCs), and Langerhans cells (LCs) show considerable transcriptional overlap and a common ontogenetic origin with Mphs (12–15). This raised doubts whether the name “DC” is still correct. On the other hand, there are also data showing mouse and human LC hierarchical clustering with subsets of conventional DCs (cDCs) (16). Here, we recall several aspects of the biology of these cell types, and we suggest to retain their name, not least for historic reasons allowing a better online search on a cell type. For example, follicular DC and pDC neither share overlaps in transcriptional profiling with cDCs nor do they exert typical DC functions. Yet, there is no discussion that they are both called DCs. In fact, human and mouse pDC share less transcriptional overlap with cDCs as compared with LC and MoDCs (17, 18), while follicular DCs are derived from marginal reticular cells, a population of mesenchymal stroma cells lining the lymph node subcapsular sinus (19). Evidence suggests that a subset of pDCs may derive from common lymphoid progenitors (CLP) and not from a common myeloid progenitor (CMP) (20) or might even derive from an own lineage (21) Nevertheless, all these cells retain their name “DC” independent from either their function or their origin from CMP, CLP, CDP (common DC progenitor), or even from non-hematopoietic progenitors.

**Figure 1:**
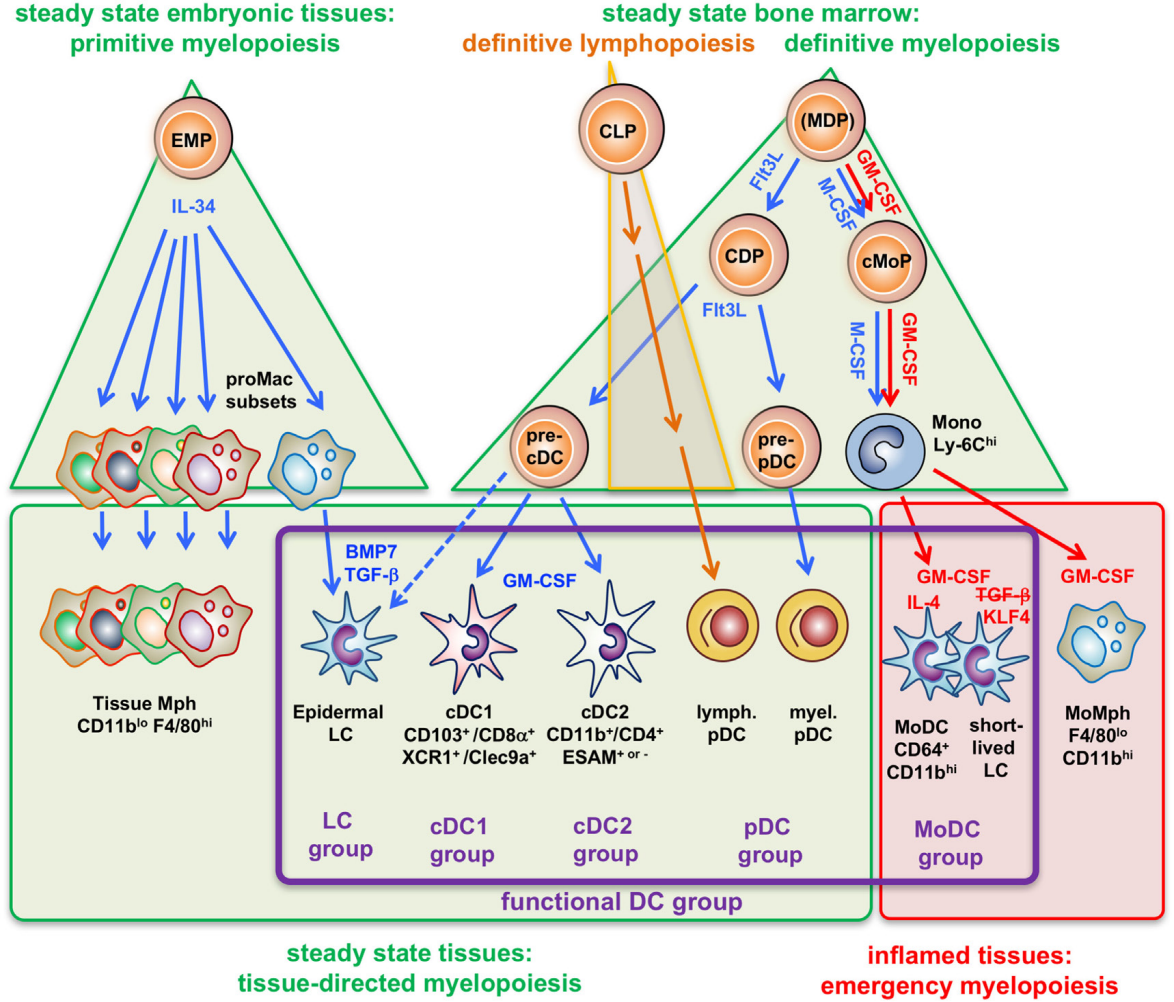
Four types of myelopoiesis and local cytokines control steady-state and inflammatory generation of grouped dendritic cell (DC) types. Early embryonic macrophage-erythrocyte precursors (EMPs) in the yolk sac and fetal liver responding to IL-34 develop into different preformed macrophage (Mph) progenitors (proMac) that generate most Mph populations and the epidermal DC subset, called Langerhans cell (LCs), which persist there throughout adulthood. As an example, differentiation of LCs requires additional cytokines such as TGF-β or BMP7 produced within the epidermis to reach the final stage of tissue-directed myelopoiesis. This primitive hematopoiesis is substituted by the definitive hematopoiesis in the BM in the adult. There, under steady-state conditions the growth factor Flt3L promotes the development of myeloid precursors giving rise to macrophages and DCs (MDPs) into common DC precursors (CDPs) that split into pre-pDCs (22) and pre-cDCs released into the blood. There is currently not yet full consensus about the potential of the cells designated as “MDPs” (2, 23, 24). Upon migration into the spleen (or other lymphatic tissues) or peripheral non-lymphatic tissues the CD103^+^ or CD8α^+^ cDC1 groups and CD11b^+^ or CD4^+^ cDC2 groups further acquire different phenotypes by tissue-derived factors, e.g., GM-CSF (in blue). By contrast, (MDPs) sensing M-CSF under steady-state conditions will develop into common monocyte precursors (cMoPs) that predominantly develop into classical Ly-6C^hi^ monocytes found in the murine blood. Under inflammatory conditions, activated CD4^+^ T-helper cells produce large amounts of GM-CSF (red) at systemic levels, thereby initiating so-called emergency myelopoiesis (25) driving MDPs and cMoPs into cell cycle and releasing increased amounts of classical monocytes into the blood. After extravasation, these monocytes can differentiate into inflammatory types of MoMph or, in the additional presence of IL-4 (red), into cells of the monocyte-derived DC (MoDC) group. Thus, differential developmental pathways merge in the generation of functional DCs (functional DC group, violet frame), independent from their origin.

To restructure the DC nomenclature on the basis of ontogenetic data alone may be highly confusing since two names or designations for the same cell will be perpetuated. For example, LCs would appear in older literature as “epidermal DCs” and newly “epidermal Mphs.” Here, we review LCs as well as GM-CSF-dependent DC and Mph generation from BM or monocytes and their functions. We believe that that functional aspects can and should be integrated in the definition of DCs (Figure 1).

## GM-CSF in DC Growth, Survival, and Functional Activation

GM-CSF supplemented BM-DC cultures for murine DC generation are under debate for their usefulness to study DC biology, mainly since Mphs and neutrophils are generated by the same cytokine in these cultures. Similarly, human MoDCs may more closely mimic Mphs rather than DCs. Factors promoting DC versus Mph development from monocytes and myeloid progenitor cells have been reported. Human monocytes differentiate toward Mphs upon exposure to IL-6, which upregulated the M-CSF receptor (M-CSFR) expression to enable consumption of their autocrine M-CSF (26). Human MoDCs can be selectively generated from monocytes in cultures by combined use of GM-CSF and IL-4 (27, 28) by inducing TNF-converting enzyme (TACE) that cleaves the M-CSFR thereby disabling autocrine M-CSF-dependent Mph generation (29). IL-4 imprinted differential epigenetic signatures for both DCs and Mphs influencing their further response to LPS (30, 31). Addition of TNF can further stabilize GM-CSF/IL-4 mediated DC skewing (32). MoDC development from monocytes is characterized by specific epigenetic programming such as histone H4K16 acetylation that was not observed in monocytes or Mphs (10). Thus, M-CSF/IL-6 promotes Mph growth while GM-CSF/IL-4 suppresses M-CSF signals and thereby support DC development.

Murine BM cells cultured with GM-CSF contain neutrophils for up to 5 days before they die and finally only loosely adherent MHC II^low^ cells and strongly adherent MHC II^neg^ Mphs remain (33–35). The MHC II^low^ cells are composed of immature DCs with the potential to become mature DCs and Mph progenitors developing into MHC II^neg^ Mphs (36, 37). Unlike for human MoDC cultures, the addition of IL-4 to murine BM-DCs does not prevent Mph growth but fulfills other functions reviewed elsewhere (38). Reversely, the generation of human BM-DCs with GM-CSF with or without IL-4 can be used to generate immunogenic or tolerogenic DCs similar as found in murine settings (39–42). Comparative analyses showed that murine BM-DCs and human MoDCs are highly similar and therefore can be considered as functional homologs (43, 44). BM-DC cultures contain proliferating cells (35). The proliferating cells mostly represent macrophage-DC progenitors (MDP) and common monocyte progenitors while differentiated Ly-6C^high^ monocytes fail to proliferate in GM-CSF cultures and therefore do not contribute substantially to the BM-DC progeny (37) and as observed in mice under steady-state conditions (45). The lack of proliferation has been described for human CD14^+^ monocytes undergoing MoDC differentiation (46).

**Figure 2:**
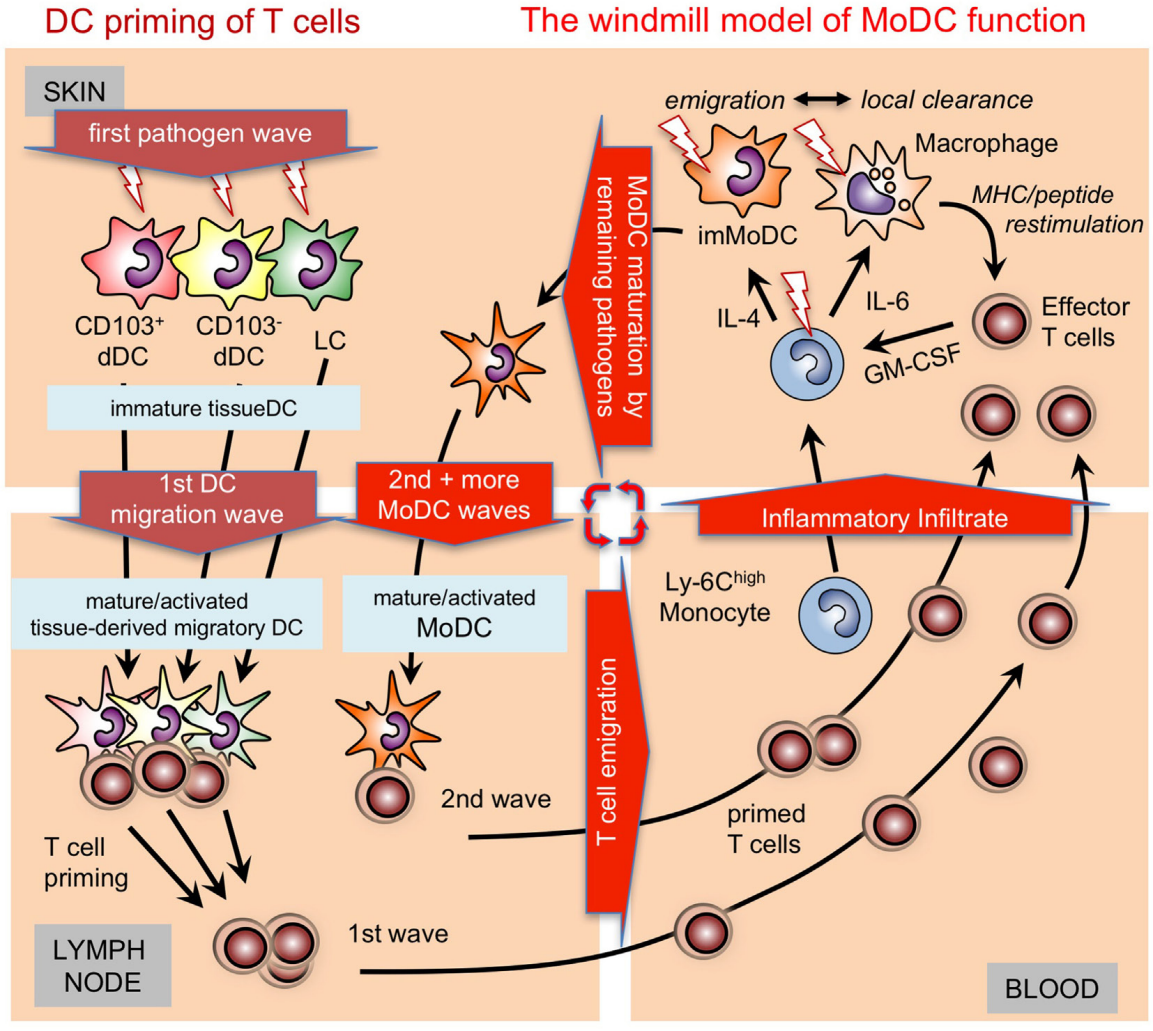
Time-dependent activation/maturation of tissue dendritic cells (DCs) and perpetuated generation of monocyte-derived DCs (MoDCs). A model showing cooperation of preexisting tissue DCs with newly generated MoDCs from infiltrating monocytes as shown before (55, 56) in a windmill-like schematic manner. Initial pathogens invading the skin as depicted here will first encounter epidermal Langerhans cell (LCs) and dermal DC subsets (dDCs). All these DC subsets are capable of capturing pathogens, undergoing maturation and can migrate CCR7 dependent into the draining lymph nodes to initiate T cell priming. The first wave of T cells will arrive together with monocytes and other cells of the inflammatory infiltrate in the infected skin. Local pathogen-specific MHC/peptide dependent reactivation of T cells, e.g., by resident or infiltrating macrophages will lead to their GM-CSF release and, together with cytokines in the environment, promote MoDC generation from monocytes. The resulting immature MoDCs follow the tissue DCs into the lymph node to perpetuate T cell priming in secondary and subsequent waves. Since the local reconstitution of emigrated tissue DCs is slow, MoDC generation by T cell-derived GM-CSF is continued as long as the infection persists as depicted graphically as a windmill model, i.e., as long as the “pathogen wind blows.”

Murine BM-DCs are typically generated *in vitro* with GM-CSF. However, protocols are available that employ Flt3L instead of GM-CSF to generate bulk populations containing mixtures of CD103^+^ cDCs, CD11b^+^ cDCs, and pDCs from mouse bone marrow (47–49) or similarly but less well defined from human peripheral blood (50, 51). The generation of such DC subtypes *in vitro* is similar to what is observed *in vivo* (Figure 2). Two articles nicely dissected the precursors of human pDCs and CD1c^+^ cDCs as well as CD141^+^ cDC and claimed to provide a method to selectively generate all three cell types from CD34^+^ progenitors (52, 53). Already earlier, a protocol for the bulk generation of all three human cDC subsets had been reported also using CD34^+^ cells (54).

A massive expansion of monocyte and dendritic cell progenitor (MDP), but very low effects on common DC progenitors (CDPs), have been found in GM-CSF supplemented BM-DC cultures (37), confirming major effects of GM-CSF on myelomonocytic cells rather than committed DC precursors (CDP) developing into Zbtb46 expressing cDCs. Although the transcription factor Zbtb46 had been considered to be specific for cDCs (57), recent data indicate that LCs co-express Zbtb46 in addition to the Mph-specific transcription factor KLF4 (58). Moreover, Ly-6C^hi^TREML4^neg^ monocytes can differentiate into Zbtb46^+^ MoDCs in response to GM-CSF and IL-4. This occurred independent of Batf3 but dependent on Irf4 and although IL-4 induced both transcription factors in murine MoDCs (59). Thus, the so far DC subset-specific transcription factors may not be restricted to a DC subset defined by ontogeny but induced by environmental cytokine signals or factors inducing specific functional activation.

However, GM-CSF has a major impact on the steady-state cDC generation from preDCs *in vivo* since mice deficient for the GM-CSF receptor (*csf2r*) have severe deficits for both subsets of cDCs (60). This is partially in agreement with the finding that also the selective *in vitro* generation of murine CD103^+^Clec9A^+^XCR1^+^ cDCs from BM cells with Flt3L was enabled by addition of only very low doses of GM-CSF (61). Moreover, CD8^+^ T cell activation during lung infection was abrogated in *csf2r^−/−^* mice (60). In fact, some data indicate that Flt3L alone may not be sufficient to generate fully functional cDCs. Functional studies with human *in vitro*-generated cDC subsets on allogeneic T cell proliferation and cross-priming obtained only poor stimulatory effects (54). Murine immature Flt3L-generated DCs were unable to induce T cell anergy or convert regulatory T cells as compared with immature GM-CSF-generated DCs (49). Additional GM-CSF signals were required by Flt3L cultured cDCs from murine BM to acquire cross-presenting capacities, which was associated but not functionally linked with the further upregulation of CD103 and an increase the frequency of CD103^+^ cells in culture (62), or CD8α^+^ and CD8α^−^ cDCs from spleen to upregulate costimulatory molecules and cytokines after activation by pathogens (63), a process presumably regulated by the STAT5 target gene cytokine inducible SH2-domain protein (CISH) (64). GM-CSF also contributes to pDC maturation/activation by inducing PU.1 dependent MHC II upregulation in pDCs (65). However, GM-CSF (with or without TNF) impaired Flt3L-induced pDC generation from murine myeloid progenitors in favor of myeloid DCs and Mphs (66).

Together, the functional data available indicate that *in vitro* GM-CSF cultures of BM cells or monocytes generate different myeloid cell types and among them a fraction clearly shows characteristics of DCs. Additional cytokine use or specific culturing/harvesting procedures further support the selective yield of DCs. Moreover, GM-CSF also controls some cDC and pDC functions.

## Heterogeneity of BM-DC Cultures

BM harbors heterogeneous cellular sources of different developmental stages of cell types including myeloid cells responsive to GM-CSF. Accordingly, the *in vitro* exposure of BM cells to GM-CSF generates different waves of BM-DC development. This can be demonstrated by culturing specific early and late myeloid precursors with GM-CSF and measuring the time required to develop into CD11c^+^ DCs (37, 67). BM precursors for two different DC subsets and Mphs responding to GM-CSF differ in their endocytosis capacities and expression of the surface markers E-cadherin, scavenger receptor A (2F8, CD204), CD11b, and Gr-1. The sorted cells with an endocytosis^high^MHC II^low^ profile gave rise either exclusively to one subset of MHC II^high^ DCs, while endocytosis^low^MHC II^low^ cells resulted in two populations, one upregulating and one downregulating MHC II molecules after two further days of culture in GM-CSF, indicative for further development into another DC subset and Mphs, respectively (36). Stimulating sorted MHC II^low^ cells with LPS further indicated that some MHC II^low^ cells within this population turned into MHC II^neg^ and adherent Mphs, while other MHC II^low^ cells (immature DCs) matured into MHC II^high^ DCs (36). Thus, these findings indicate that bulk cultures as well as MHC II^low^ cells generated in response to GM-CSF are not uniform. Rather they include cells with differentiation potential for Mphs and at least two different subsets of immature DCs. Since such an MHC II^low^ Mph/DC mix will blur the results of mRNA profiling for cellular subset identification careful dissection of all subsets is a prerequisite. Additional factors may bias results from murine BM-DCs, such as components in the culture medium or fetal calf serum which can influence the outgrowth of DCs (68). While maturation of isolated murine LCs was not influenced under serum-free conditions (69), serum-free BM cell cultures failed to generate fully functional murine BM-DCs (70). BM-DC heterogeneity has also been reported by others (37) and similarly human DC/LC cultures supplemented with GM-CSF are reportedly heterogenous (71). These phenotypically distinct *in vitro*-generated DC subsets now require more detailed -omics profiling but importantly, also distinguishing between immature/resting and mature/activated stages. DCs expressing different levels of MHC II- and costimulatory molecules will certainly differ in transcriptional patterns although belonging to the same ontogenetic DC subset (72). Such data allow then further comparisons with the same maturation stages of Flt3L *in vitro*-generated or *ex vivo*-isolated cDC and pDC subsets.

Since some DC subsets share their capacities for pathogen recognition (73, 74), they also may translate them into same polarized pathogen-specific T-helper cell (Th) response. The treatment of bulk BM-DC cultures with different maturation stimuli appeared valuable to determine distinct response patterns by mRNA analyses, which could be attributed to DC-mediated Th1 and Th2 polarization (75).

## Monocytes as a Source for DCs

Under steady-state conditions, MoDCs are hardly found in mice and man (76). However, epithelia and mucosal tissues do contain detectable amounts of MoDCs presumably induced by commensals (77). Clearly, inflammatory and infectious conditions can recruit Ly-6C^high^ monocytes into tissues, which then develop into DCs that initiate T cell priming in the draining lymph nodes (55, 78–81). Similar results were obtained for the appearance of MoDCs in humans from synovia of rheumatoid arthritis patients and ascites from cancer patients (44). Thus, fully functional MoDCs can be differentiated under inflammatory or infectious conditions from monocytes in mice and humans.

In the absence of or early after depletion of Batf3-dependent CD103^+^ DCs in C57BL/6 mice with *Leishmania* infection a Th1 response does not develop (82). However, during later stages 4 weeks after infection, dermal MoDCs were the only DC subset at the site of skin infection and in the draining lymph nodes to present *Leishmania* antigens and to produce IL-12 to maintain the Th1 response (56). Together, these data led us to establish a “windmill model” of MoDC function (Figure 2).

*In vitro* studies showed a strict dependency of murine BM-DCs and human MoDCs on GM-CSF. When such *in vitro*-generated cells were injected s.c. or i.d. they homed to the T cell areas of the draining lymph nodes (83), a typical DC function that requires CCR7 expression (84). In comparison, inert particles (85) were flushed into the lymph nodes by the lymph fluid; they only penetrated the subcapsular sinus but did not reach the T cell zone. From these studies, it had been concluded that GM-CSF is also critical for driving inflammatory MoDC generation *in vivo*.

Surprisingly, abrogation of GM-CSF or its receptor in mice did not affect MoDC generation and activation of CD8^+^ T cell responses. Conversely, deficiency of the M-CSFR (*csf1*) impaired inflammatory MoDC recruitment and CD80/CD86 surface expression (60). Therefore, the role of GM-CSF for the generation of inflammatory MoDC *in vivo* remains questionable. On the other hand, Mphs but not DCs arise from human monocytes in response to M-CSF or IL-34 *in vitro* (86), the latter representing a recently discovered new ligand for the M-CSFR. Thus, the precise roles of M-CSF and GM-CSF for MoDC generation *in vitro* and *in vivo* are not fully understood and additional factors from the local inflammatory environment may critically contribute to the monocyte-to-DC conversion.

## *In Vitro*-Generated MoDCs as Models for *In Vivo* cDC Function?

Human and mouse MoDCs can be generated from blood or BM monocytic cells and precursors in large amounts. This has enabled early studies to investigate them by biochemical techniques. However, one should be careful to simply extrapolate these functional analyses generalized from MoDCs to cDCs. The different cDCs subsets appear to have specialized preferences for cross-presentation (CD8α^+^/CD103^+^ cDC1) or MHC II dependent presentation (CD4^+^/CD11b^+^ cDC2) (87). More recent studies further dissected cDC2 into ESAM^+^ cDC2 inducing Th17 and ESAM^+^ cDC2 specialized to promote Th2 polarization (5). By contrast, *in vitro*-generated MoDCs (BM-DCs) appear more versatile in their functional adaptation to generate specific Th1, Th2, and Th17 responses *in vitro* or after injection into mice depending on their stimulation with LPS or TNF (75), cholera toxin (88), or in cross-presenting antigens to CD8^+^ T cells (89) or presenting glycolipids on CD1d molecules for polarizing NKT cells into either IFN-γ or IL-4 producing subtypes (90, 91). However, indirect cross-priming and NKT cell priming by injected BM-DCs with endogenous spleen DC subsets has been observed (92), which may point to DC–DC cooperation as observed in lymph nodes after virus infection (93).

Several early biochemical findings with MoDCs/BM-DCs could not be confirmed using more recent mouse models for cDCs *in vivo*. This includes the role of the transcription factor CIITA for the regulation of MHC II genes and DC development (94) and the transcriptional regulation of cross-presentation (59, 95). Clearly, MoDC functions should not be merely extrapolated to cDC functions. Nevertheless, if sensibly used and in a critical manner, these cells certainly retain value for studying certain aspects of DC biology.

## GM-CSF-Generated MoDC as Tumor Vaccines

GM-CSF/IL-4 generated MoDCs *in vitro*, or the direct use of GM-CSF as adjuvant to promote MoDC generation *in situ*, remain both promising concepts in antitumor vaccine trials (96–98), especially in potential future combinations with modern “immune checkpoint inhibitors” such as anti-CTLA-4 or anti-PD-1 (99, 100). MoDC treatment of melanoma appears highly successful in melanoma patients and showed the same 19% 12 year survival rate as compared with achieved by ipilimumab treatment (101). Under steady-state conditions monocytes rarely immigrate into peripheral tissues to develop into MoDCs due to the absence of inflammation and high GM-CSF concentrations in the local environment (76). Thus, efficient monocyte immigration and their conversion into MoDC allowing migration from peripheral tissues or injection sites into the lymph node may benefit from an inflammatory environment (Figure 2). Preinjection of the DC injection site with TNF or repetitive DC injections into the same site have been shown to dramatically improve DC homing in mice by upregulating CCR7 on DCs and also its ligand CCL21 in lymphatic endothelial cells (102). In a recent clinical study of DC vaccination in glioblastoma patients, the injection site was pretreated with tetanus/diphtheria toxoid before injection of MoDCs, which dramatically improved vaccine efficacy as observed by the patients’ survival rate (103). The benefits of low but not high doses of local GM-CSF as an adjuvant have also been elaborated (104). It would be interesting to investigate the functional role of GM-CSF produced at immunization sites by infiltrating T cells both to enhance cDC responsiveness and maturation (63) as well as in the local conversion of monocytes into MoDCs.

## Epidermal LCs as DCs

Recently, it was found that LCs were derived from EMC precursors in the yolk sac by stimulation of the M-CSFR through IL-34 (105–107) together with various tissue Mph populations (108–110). This led to a frequently expressed notion that LCs represent tissue-resident Mphs. However, functionally LCs also show a strong overlap with DCs since they are migratory both in the steady state to induce tolerance and during inflammation. They capture selective pathogens and migrate to initiate T cell responses in the lymph nodes (6, 111, 112). Moreover, microglia and in part LC are the only yolk sac-derived cells, and all other early Mph populations are generated in the aorta-gonad-mesonephros and fetal liver (113), thus further challenging the pure Mph identity of microglia and LCs. Taken together, LCs were found to share both characteristics of Mphs and DCs (6, 58).

It remains to be solved, whether a further subset division into Mph-like LCs and DC-like LCs may exist, because under infectious or inflammatory conditions never all LCs emigrate from the epidermis, even under harsh conditions. Evidence for a “dual identity” of LCs was recently provided from isolated murine bulk LCs expressing both the Mph-specific transcription factor Mafb and the transcription factor Zbtb46 specific for cDCs (58). In the future, single cell mRNA sequencing may reveal whether both factors are expressed in the same cells or in different LC subsets.

Under inflammatory conditions, blood monocytes do replenish the local pool of epidermal and mucosal LCs to compensate for the loss of LCs that emigrated toward the lymph node (114–116) (Figure 2). In the case of the murine oral cavity, a steady-state population of cDC and monocyte-derived LCs have also been described (116). Murine monocyte-derived LCs enabled an Id2-independent short-term reconstitution of the epidermal LC pool (115). Similarly, human CD14^+^ monocytes can be induced to acquire LC phenotypic characteristics *in vitro*. LC phenotype induction required TGF-β1, GM-CSF, and IL-4 (117). Later, it was shown that a combination of TGF-β, GM-CSF, and Notch ligand (Delta-1 Jagged2) allows efficient generation of LC-like cells from monocytes (118, 119). Whether monocytes take over long-term reconstitution of epidermal LCs remains unclear (115, 120, 121) According to murine studies, monocytes may only transiently replenish LC-like cells exhibiting maintained expression of monocyte markers and reduced expression of LC markers. Conversely, another so far undefined BM-derived precursor may lead to a long lasting replenishment of LCs in an Id2-dependent manner (115). Human CD1c^+^ circulating peripheral blood DCs rapidly acquired LC characteristics *in vitro*, and these cells did not require Notch ligand for LC differentiation (122, 123). Whether these *in vitro*-generated candidate non-monocyte LC precursors replenish human LCs *in vivo* remains unknown (124, 125). It must be considered that human LCs lack Mph-associated markers and cross-species transcriptional analyses of skin DC subsets revealed that human LCs are more closely related to human and murine DCs rather than to murine LC, the latter exhibiting Mph markers (126). We recently observed that CD14^+^ human blood monocytes lose expression of the transcription factor KLF4 during LC commitment (in response to GM-CSF/TGF-β and Notch ligand), and loss of KLF4 is accompanied by loss of monocyte markers. Moreover, loss of KLF4 may represent a prerequisite for TGF-β1-mediated induction of RUNX3, a master transcription factor inducing LC lineage commitment (127). Given that KLF4 restores monocyte/Mph differentiation from fetal liver progenitor cells lacking PU.1 together with driving human monocyte differentiation, KLF4 can be considered a lineage identity factor for monocytes/Mphs (128). Consistently, abovementioned murine short-term LCs (Id2-independent) expressed KLF4, whereas long-term LCs (Id2-dependent) lacked KLF4 (115). Although the earliest LC precursors develop in parallel with embryonic Mphs and only differentiate into the LC phenotype within the epidermis, they specifically acquire typical DC transcription factors. Ahr and Runx3, which are not shared with any other Mph subset (15) have been identified in DCs before, e.g., Ahr in BM-DCs and splenic DCs (129–131) and Runx3 in splenic Esam^hi^CD11b^+^ cDCs (132). Also at a global transcriptional level human and murine LC resemble more closely cDCs than monocytes or monocyte-derived cells (16). Thus, it appears that a cell of mononuclear phagocyte ontogeny and identity can convert into epidermal DCs, called LCs (Figure 1).

Mo-derived (or CD1c^+^ blood DC-derived) LCs and DCs may fulfill an important task to replenish tissues or compartments after the resident cDCs have migrated out of the tissue as illustrated by the “windmill model” of MoDC function (Figure 2). Tissue-resident or blood precursors both contribute to reconstitute LC in the murine or human system (122, 123, 133). The local potential for replenishment may be limited especially under chronic inflammatory conditions. In such a situation GM-CSF-driven emergency myelopoiesis in the BM and monocyte conversion to MoDCs in inflamed or infected peripheral tissues is required for replenishment with DCs. Although not directly tested, both populations are assumed to fulfill the same functions in immune surveillance in spite of being derived from different lineage origins.

The huge body of data that has been acquired using these models and omics data has critically contributed to our current profound knowledge on DC biology. Clearly, there is a need for clarification and simplification of DC nomenclature. However, it is important to take into account all available knowledge—new and old—when attempting to design a new DC nomenclature to avoid uncertainty and irritation in the field. This applies even more so to LCs. It was LCs that, for the first time, allowed to perceive and observe the concept of DC maturation (134), a true hallmark of the DC nature. Thirty years later, DC maturation is understood in much more detail (135). Also, we have come to realize that LCs can also induce tolerance, depending on the circumstances and the quality of maturation (136–138). Successful approaches to employ their DC-like migratory and T cell priming potential for vaccine technology may underscore their DC-like functions (139, 140). Thus, the LC example emphasizes the importance to consider also functional lineage plasticity besides ontogenetic data.

## Evidence for DCs Arising from Emergency Granulopoiesis

Neutrophilic granulocytes represent by far the most abundant leukocyte subtype in human bone marrow. They arise from hematopoietic stem cells via granulocyte/macrophage progenitor cells, a cell stage hierarchically upstream of macrophage/DC progenitors (MDP). Steady-state BM contains various differentiation stages of neutrophils. In response to acute inflammation or trauma, neutrophils are rapidly mobilized from bone marrow into peripheral blood to meet the bodies’ high demand on these cells to fight microbial infections. These emergency neutrophils mainly exhibit a band-shaped nucleus and differ from polymorphonuclear neutrophils (PMN) observed in the steady state. Such neo-recruited neutrophils in human peripheral blood were shown to possess *in vitro* DC differentiation potential in response to 5- to 9-day culture with GM-CSF, IL-4, and TNF, a process occurring without cell proliferation (141). While most generated cells lacked neutrophil markers they still expressed myeloperoxidase, a lysosomal protein found in granulopoietic cells and blood monocytes. Interestingly, so-called “neutrophil-DC hybrids” were generated from murine bone marrow in response to GM-CSF (142) and appeared *in vivo* in experimentally induced inflammatory lesions in mice (143). In line with this, neutrophils from G-CSF mobilized blood “trans”-differentiated into monocytic cells *in vivo* in mice or for human cells *in vitro* in response to GM-CSF, TNF, IL-1β (144) or GM-CSF, M-CSF, TNF, IL-4, IFN-γ (145). This neutrophil plasticity seems to be confined to immature neutrophils characterized by their band-like-shaped nuclei, since PMN from the blood of healthy individuals lacked monocyte or DC differentiation potential (141, 144). Inflammatory signals encountered within lesions may transcriptionally reprogram neutrophils into monocytes/Mphs given that the selective activation of p38MAPK (induced by inflammatory signals) was sufficient to induce monocyte differentiation from granulocytic cells (144). Therefore, left-shifted band-stage neutrophilic granulocytes seem to possess a potential to differentiate into cells of the mononuclear phagocyte system including DCs rapidly within inflammatory lesions [recently reviewed in Ref. (146)]. Although this granulopoietic pathway for DCs still remains poorly defined, to term the resulting monocytes and DCs still “neutrophils,” only based on their ontogeny, would be misleading.

## Conclusion

Bone marrow-derived DC, MoDC, and LC share ontogenic and transcriptional similarities with the Mph lineage but also with cDCs. They possess strong phenotypes and functions as known for cDCs (2). Further dissection of the functional plasticity of monocytes in their acquisition of DC-like or Mph-like functions is required. A hematopoietic model including ontogenetic and functional DC/Mph differences is proposed here (Figure 1). Recent data focusing on steady-state distributions of DC subsets and DC *in vivo* function with respect to defined cytokine deficiencies (147) or the interplay between GM-CSF, M-CSF, and IL-3 (60) may point to alternative approaches toward a better understanding of GM-CSF-derived cells. In any case, the purity of DC populations defined on the basis of all published data as well as differences in activation/maturation stages have to be considered to obtain meaningful RNA sequencing data on DC subsets. Why GM-CSF-based protocols are so successful for generation of murine and human MoDCs *in vitro*, but GM-CSF-deficient mice (60) or GM-CSF injected mice (148) fail to show such a role requires further investigation. Together, based on these considerations, we propose that a nomenclature for DCs and Mphs may benefit from considering all available and therefore also functional characteristics of cells in addition to their developmental or hematopoietic origination.

## Author Contributions

All authors listed have a made substantial, direct and intellectual contribution to the work, and approved it for publication.

## Conflict of Interest Statement

The authors declare that the research was conducted in the absence of any commercial or financial relationships that could be construed as a potential conflict of interest.
